# *Glaesserella parasuis* serotype 4 exploits fibronectin via RlpA for tracheal colonization following porcine circovirus type 2 infection

**DOI:** 10.1371/journal.ppat.1012513

**Published:** 2024-09-12

**Authors:** Mengru Guo, Yuhui Li, Jinsheng Tang, Qing Wang, Qiancheng Wang, Hong Zhou, Huixing Lin, Zhe Ma, Hongjie Fan

**Affiliations:** 1 MOE Joint International Research Laboratory of Animal Health and Food Safety, College of Veterinary Medicine, Nanjing Agricultural University, Nanjing, China; 2 Anhui Province Key Laboratory of Veterinary Pathobiology and Disease Control, College of Animal Science and Technology, Anhui Agricultural University, Hefei, China; 3 College of Animal Science, Anhui Science and Technology University, Fengyang, China; Medical College of Wisconsin, UNITED STATES OF AMERICA

## Abstract

Porcine circovirus type 2 (PCV2) often causes disease through coinfection with other bacterial pathogens, including *Glaesserella parasuis* (*G*. *parasuis*), which causes high morbidity and mortality, but the role played by PCV2 and bacterial and host factors contributing to this process have not been defined. Bacterial attachment is assumed to occur via specific receptor-ligand interactions between adhesins on the bacterial cell and host proteins adsorbed to the implant surface. Mass spectrometry (MS) analysis of PCV2-infected swine tracheal epithelial cells (STEC) revealed that the expression of Extracellular matrix protein (ECM) Fibronectin (Fn) increased significantly on the infected cells surface. Importantly, efficient *G*. *parasuis* serotype 4 (GPS4) adherence to STECs was imparted by interactions with Fn. Furthermore, abrogation of adherence was gained by genetic knockout of Fn, Fn and Integrin β1 antibody blocking. Fn is frequently exploited as a receptor for bacterial pathogens. To explore the GPS4 adhesin that interacts with Fn, recombinant Fn N-terminal type I and type II domains were incubated with GPS4, and the interacting proteins were pulled down for MS analysis. Here, we show that rare lipoprotein A (RlpA) directly interacts with host Fibronectin mediating GPS4 adhesion. Finally, we found that PCV2-induced Fibronectin expression and adherence of GPS4 were prevented significantly by TGF-β signaling pathway inhibitor SB431542. Our data suggest the RlpA-Fn interaction to be a potentially promising novel therapeutic target to combat PCV2 and GPS4 coinfection.

## Author summary

Porcine circovirus type 2 and *Glaesserella parasuis* (*G*. *parasuis*) are common upper respiratory tract pathogens in pigs, which co-infection causes high morbidity and mortality. However, the underlying mechanisms of host-pathogen interactions during PCV2 and GPS4 co-infection remain unclear. Here, we focused on the interaction of PCV2-infected cells and GPS4, which revealed that swine tracheal epithelial cells (STEC) infected with PCV2 exhibit the increased expression of Fn. Notably, PCV2 infection-induced Fn interacting with RlpA was shown to impart efficient adherence to GPS4. Fn and Integrin β1 antibody blocking and SB431542 counteracted PCV2 infection-induced GPS4 colonization. These studies reveal a novel receptor-ligand interaction that enhances colonization of the tracheal by GPS4 following PCV2 infection and highlights the importance of Fn-RlpA in host-pathogen interactions. In addition, RlpA was discovered for the first time as an adhesin of GPS4, which provided a new idea for the development of *G*. *parasuis* vaccines.

## Introduction

Porcine circovirus type 2 (PCV2) is considered the main pathogen of porcine circovirus-associated diseases (PCVAD) [1]. Most of the time, the infection is subclinical, but in some circumstances, such as coinfections with other respiratory pathogens, it can cause Post-weaning Multisystemic Wasting Syndrome (PMWS), clinically characterized by wasting respiratory disease, and enteritis, such as *Streptococcus suis*, *Actinobacillus pleuropneumoniae*, *Mycoplasma hyopneumoniae*, and *Glaesserella parasuis* (*G*. *parasuis*), which are responsible for great economic losses in pig industry [2]. *G*. *parasuis* is a commensal bacterium in the upper respiratory tract of pigs, which can damage the porcine respiratory epithelial barrier and cause the swine Glässer disease, characterized by fibrinous polyserositis, pneumonia, arthritis, and meningitis [3,4]. Epidemiological investigation showed that PCV2 and *Glaesserella parasuis* serotype 4 (GPS4) co-infection is widespread in pig farms, resulting in high morbidity and mortality [5]. Our previous studies have shown that PCV2 infection causes immune dysregulation in piglets and disrupts the integrity of the tracheal epithelial barrier, which facilitates GPS4 infection, increasing the clinical symptoms of piglets [6,7]. However, the underlying mechanism of the PCV2 and GPS4 synergy leading to disease progression has yet to be discovered, thus hampering the production of effective prophylactic and therapeutic intervention options.

Extracellular matrix proteins (ECM), such as fibronectin (Fn), collagen, and laminin, interact with integrins, which transduce signals to regulate cell growth, differentiation, migration, and other cellular activities. ECM proteins and integrins are receptors that bind to microbial surface components, recognizing adhesive matrix molecules (MSCRAMM) for bacterial adherence and invasion [8,9]. The expression of these cellular adhesion molecules can be upregulated through TGF-β [10]. This cytokine is secreted as an inactive or latent protein that subsequently is activated through various mechanisms [11]. Adherence to host tissue is a critical initial step to establish infection. The most frequently observed bacteria in coinfections are *Streptococcus pneumoniae* (*S*. *pneumoniae*), group A *Streptococcus pyogenes* (GAS), *Staphylococcus aureus* (*S*. *aureus*), and *Haemophilus influenza* [12–14]. These bacteria require ECM components or integrins as receptors for adherence [15–18]. Li reported that Influenza viral neuraminidase primes bacterial coinfection through TGF-β–mediated expression of host cell receptors [19]. These findings suggest that TGF-β plays a role in IAV-enhanced bacterial adherence. However, the key host factors contributing to PCV2-triggered GPS4 coinfection remain elusive.

Rare lipoprotein A (RlpA) contains a C-terminal SPOR domain. *Pseudomonas aeruginosa* (*P*. *aeruginosa*) RlpA is a lytic transglycosylase (LT) [20] predicted to be anchored to the inner leaflet of the outer membrane [21–23]. All known proteins possessing SPOR domains are either involved in cell division or morphogenesis. For example, four proteins with SPOR domains have been identified in *Escherichia coli* (DamX, DedD, FtsN, and RlpA), all of which are involved in cell division with one (FtsN) assessed as indispensable [24,25]. Of all SPOR-domain-containing proteins, RlpA is the most highly conserved across bacterial species [20], which underscores its important physiological role in bacteria. It is predicted to have two domains: a catalytic "double-ψ β-barrel" domain (DPBB; Pfam 03330) and a C-terminal SPOR domain. RlpA septal ring localization has been shown in *E*. *coli* [24,25] and *P*. *aeruginosa* [20], and its function is needed for efficient separation of daughter cells and maintenance of rod shape in *P*. *aeruginosa* but not in *E*. *coli* [24,25]. However, whether the GPS4 outer membrane rare lipoprotein A RlpA participates in PCV2-induced bacterial co-infection is unknown.

In the present study, novel findings showing that rare lipoprotein A (RlpA), a lytic transglycosylase, is involved in the adherence of GPS4 following PCV2 infection are presented. Interactions of RlpA with Fn, whose surface expression was induced by activation of the TGF-β/Smad signaling pathway after PCV2 infection, were found to promote GPS4 adherence to PCV2-infected STECs. Also, Fn knockout, Fn and Integrin β1 antibody blocking, and SB431542 treatment counteracted PCV2 infection-induced GPS4 colonization. Our study demonstrates a novel receptor-ligand interaction that enhances tracheal colonization of GPS4 following PCV2 infection and highlights the importance of Fn-RlpA in host-pathogen interactions.

## Results

### PCV2 infection promotes the adhesion of GPS4 in STEC

**Fig 1:**
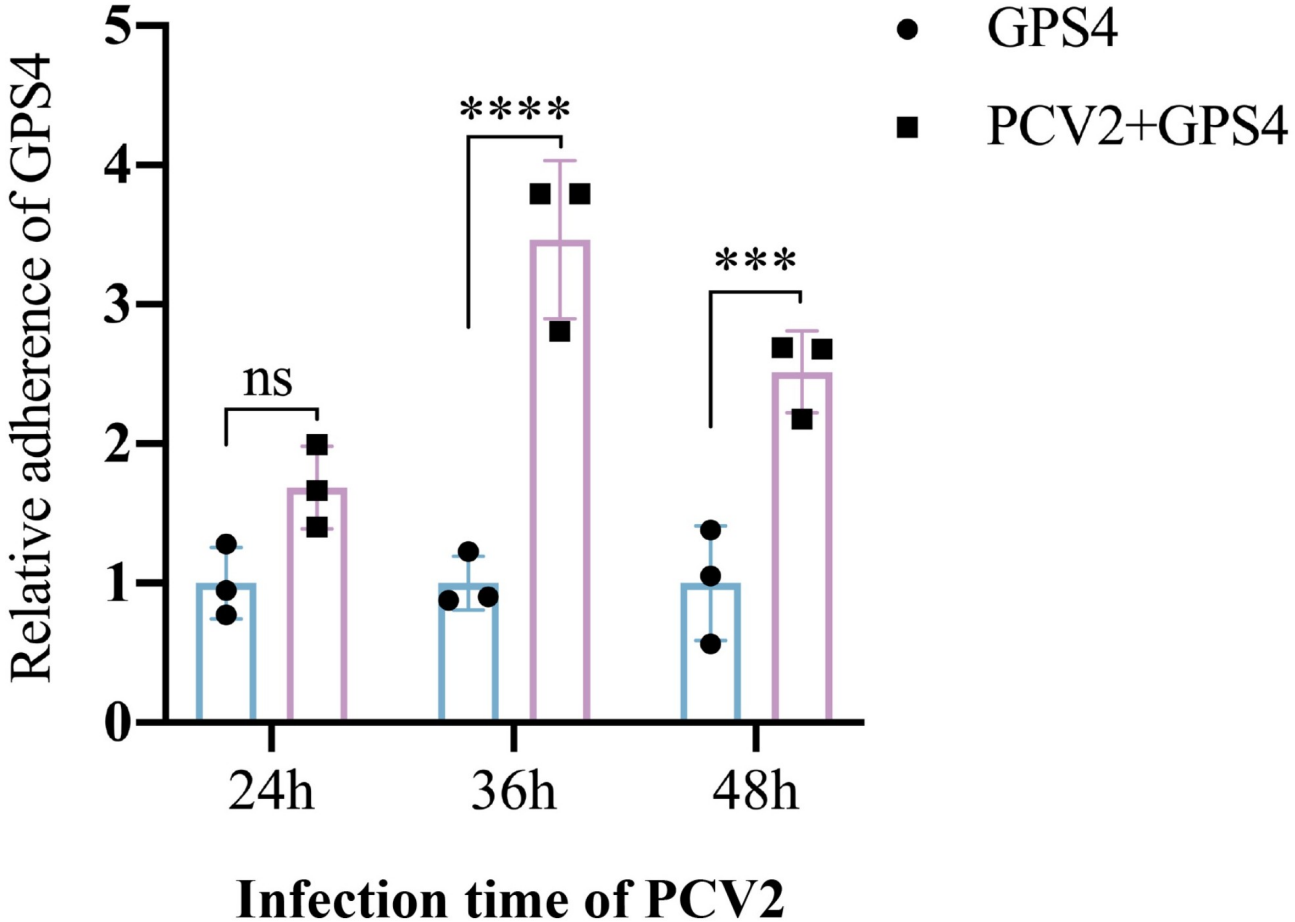
PCV2 infection promotes the adhesion of GPS4 in STEC. Adhesion of GPS4 to STEC. After PCV2 infection at 24, 36, and 48h, the cells were infected with GPS4 for another 2 h, and the number of adherent bacteria was counted. The CFUs of GPS4 in PCV2-uninfected STEC cells are considered to be 1. Results are shown as means ± SDs of three independent experiments. Statistical analysis was performed using two-way ANOVA. ***, *P* <0.001; ****, *P* < 0.0001; ns, not significant.

Epidemiological investigation showed that PCV2 and GPS4 coinfection is the most common in piglets’ post-weaning multisystemic wasting disease (PMWS) caused by PCV2, resulting in high morbidity and mortality [5]. Bacterial colonization of the upper respiratory tract is considered a prerequisite for invasive infection, leading to bacterial invasion of other tissues or spreading to the lower respiratory tract [12]. Therefore, we speculate that PCV2 infection can promote the adhesion of GPS4 to swine tracheal epithelial cells (STEC). Adhesion assays were performed to determine whether PCV2 infection promotes GPS4 adhesion to STEC cells. As shown in Fig 1, PCV2 infection promoted the adhesion of GPS4 to STEC cells compared with the GPS4 single-infection group, which was most significant at 36 h after PCV2 infection, consistent with previous preliminary experimental results [7].

### PCV2 infection upregulates the expression of extracellular matrix protein Fn

**Fig 2:**
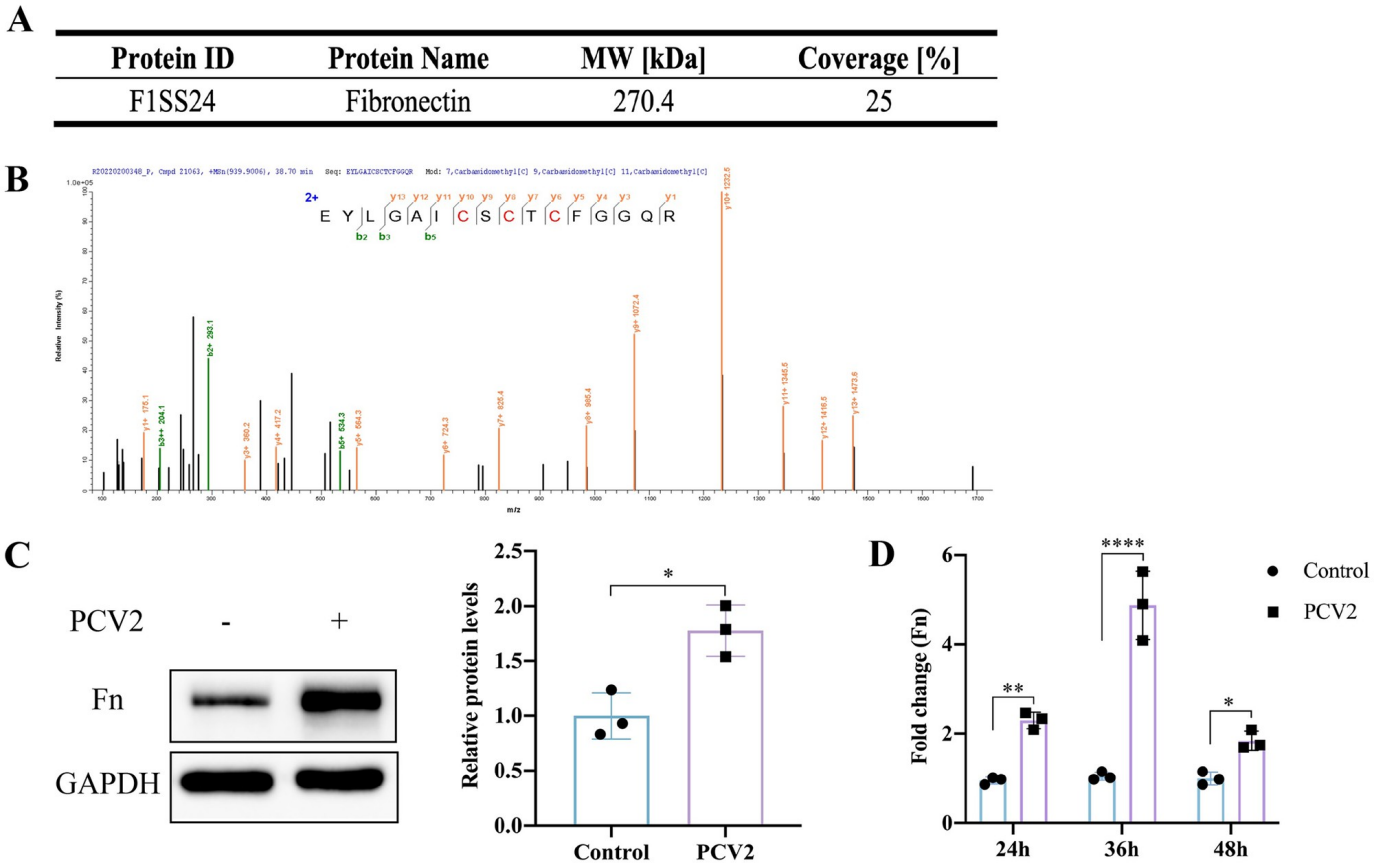
PCV2 infection induces Fn expression on tracheal epithelial cells. After PCV2 infection for 36 h, swine tracheal epithelial cell surface proteins were isolated and identified by mass spectrometry (A and B). (A) LC-MS/MS analysis result for Fn. (B) LC-MS/MS spectrum for the Fn peptide. (C) Lysates of STEC cells infected with PCV2 (1 MOI) for 36 h were analyzed by Western blot. Image J was used to analyze the density of protein bands, and GAPDH was used as an internal reference. (D) mRNA levels of Fn in STEC cells infected with PCV2 for 24, 36, and 48h were analyzed by RT-qPCR. Data are shown as means ± SDs of three independent experiments. Statistical analysis was performed using an unpaired *t*-test (C) and two-way ANOVA (D). *, *P* < 0.05; **, *P* < 0.01; ****, *P* < 0.0001.

Host inflammatory response to a viral infection leads to increased or ectopic expressions of multiple proteins that serve as host receptors for bacteria [12]. As a first step toward understanding the pathogenesis of GPS4 following PCV2 infection, we attempted to determine which proteins were increased on the surfaces of STEC cells following viral infection. STEC cells were infected with PCV2, followed by exposure to a membrane-impermeable biotinylation reagent (A44390, ThermoFisher Scientific). Cell surface proteins were then isolated using Thermo Scientific NeutrAvidin Agarose and subjected to mass spectrometry (MS) analysis, which showed several different upregulated proteins on the surfaces of PCV2-infected epithelial cells. Mass spectrometry analysis of these proteins revealed peptides corresponding to integrin binding, actin binding, muscle contraction, cell differentiation, and laminin binding-related proteins. We concentrated on the extracellular matrix protein (ECM) among the host molecules: Interacting with integrins, Fibronectin (Fn) (Fig 2A and 2B) transduces signals that control cell proliferation, differentiation, migration, and other cellular processes [19]. To further confirm the effect of PCV2 infection on Fn protein expression, STEC was infected with PCV2 for 36 h. Cell lysates were collected for Western blot assay. Western blot analysis showed that PCV2 infection can significantly induce the expression of Fn, compared with uninfected STECs (Fig 2C). In addition, STEC was infected with PCV2 for 24, 36, and 48 h, total RNA was extracted for RT-qPCR analysis. Compared with the uninfected STECs, PCV2 infection can up-regulate Fn transcription levels, which is extremely significant at 36 h after virus infection (Fig 2D). The above results show that PCV2 infection upregulates the expression of extracellular matrix protein Fn.

### PCV2 infection-induced Fn promotes GPS4 adherence

**Fig 3:**
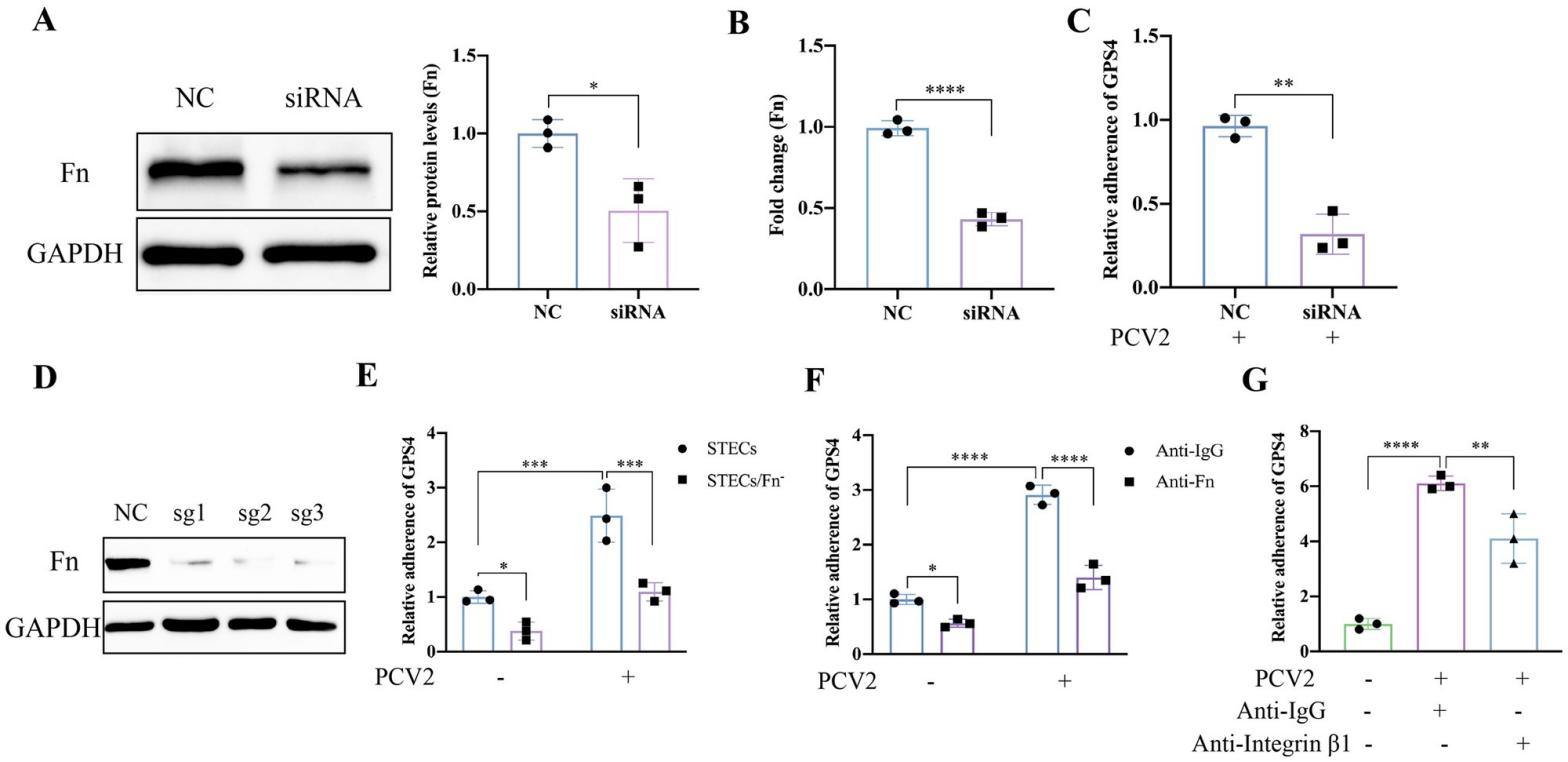
PCV2 infection-induced Fn promotes GPS4 adherence. In order to preliminarily verify whether PCV2 infection-induced Fn is associated with GPS4 adhesion, Fn was interfered with with siRNA. (A) Un-transfected and Fn siRNA-transfected STEC cells, Western blot analysis of Fn protein levels. Image J was used to analyze the density of protein bands, and GAPDH was used as an internal reference. (B) The total mRNA level of Fn was analyzed by RT-qPCR. (C) PCV2 infection un-transfected and Fn siRNA-transfected STEC cells for 36 h, GPS4 infection for another 2 h, adhesion assay was performed. To further examine whether PCV2-induced Fn promotes GPS4 adhesion, CRISPR/Cas9/sgRNA technology was performed to target the exon of the Fn gene in STEC cells specifically. (D) Western blot detects the expression of Fn in STEC infected with lentivirus, and GAPDH was used as an internal reference. (E) Adherence assays of GPS4 in STEC and STEC/Fn^-^ cells inoculated with PCV2 (MOI = 1) or DMEM for 36 h before GPS4 (MOI = 100) infection for 2 h. The colony forming units (CFUs) of GPS4 in PCV2-uninfected STEC cells are considered to be 1. (F) Colonization of GPS4 in STEC cells inoculated with PCV2 (MOI = 1) or DMEM for 36 h before treatment with rabbit polyclonal anti-Fn antibody or rabbit monoclonal anti-immunoglobulin G (IgG; 10 μg/ml) for 3 h, followed by GPS4 (MOI = 100) infection for another 2 h. (G) Colonization of GPS4 in STEC cells inoculated with PCV2 (MOI = 1) for 36 h before treatment with rabbit anti-Integrin β1 antibody or rabbit monoclonal anti-immunoglobulin G (IgG; 10 μg/ml) for 3 h, followed by GPS4 (MOI = 100) infection for another 2 h. The CFUs of GPS4 in PCV2-uninfected STEC cells are considered to be 1. Data are shown as mean ± SDs of three experiments, and Image J software was used to analyze the intensity of protein bands (A) and GAPDH as an internal reference. Statistical analysis was performed using *t*-test (A, B, and C), two-way ANOVA (E and F) and one-way ANOVA (G). *, *P* < 0.05; **, *P* < 0.01; ***, *P* <0.001; ****, *P* < 0.0001.

ECM synthesis increases for tissue repair following viral infection, and Fn, a major component of the ECM, is produced during this process. Fn is frequently exploited as a receptor for bacterial pathogens, including GAS [19], *S*. *aureus* [26], *Staphylococcus epidermidis* (*S*. *epidermidis*) [27], and *Haemophilus influenzae* (*H*. *influenzae*) [28]. To examine whether Fn serves as a receptor for GPS4, we used siRNA to interfere with Fn, and its protein expression and transcription levels decreased significantly (Fig 3A and 3B). Compared with the non-interference group, the GPS4 adherence in the siRNA interference group was significantly reduced (Fig 3C). To confirm the function of Fn further, we performed CRISPR/Cas9/sgRNA technology to target the exon of Fn gene in STEC cells specifically. The result showed high on-target efficiency of Fn (Figs S1 and 3D). To determine the role of Fn in PCV2-induced a bacterial coinfection, bacterial adherence assay was performed with GPS4 in STECs/Fn^-^. After PCV2 infection, GPS4 colony-forming units (CFUs) in STEC/Fn^-^ cells were much fewer than those in wild-type (WT) STEC cells (Fig 3E). Further, antibody-mediated blockade experiments showed that after PCV2 infection, the difference in GPS4 colonization induced by PCV2 was eliminated by cell pretreatment with anti-Fn and anti-integrin β1 antibodies (Fig 3F and 3G), indicating that PCV2 upregulated Fn had important roles during GPS4 infection.

### GPS4 adheres to tracheal epithelial cells through the interaction of RlpA with Fn

**Fig 4:**
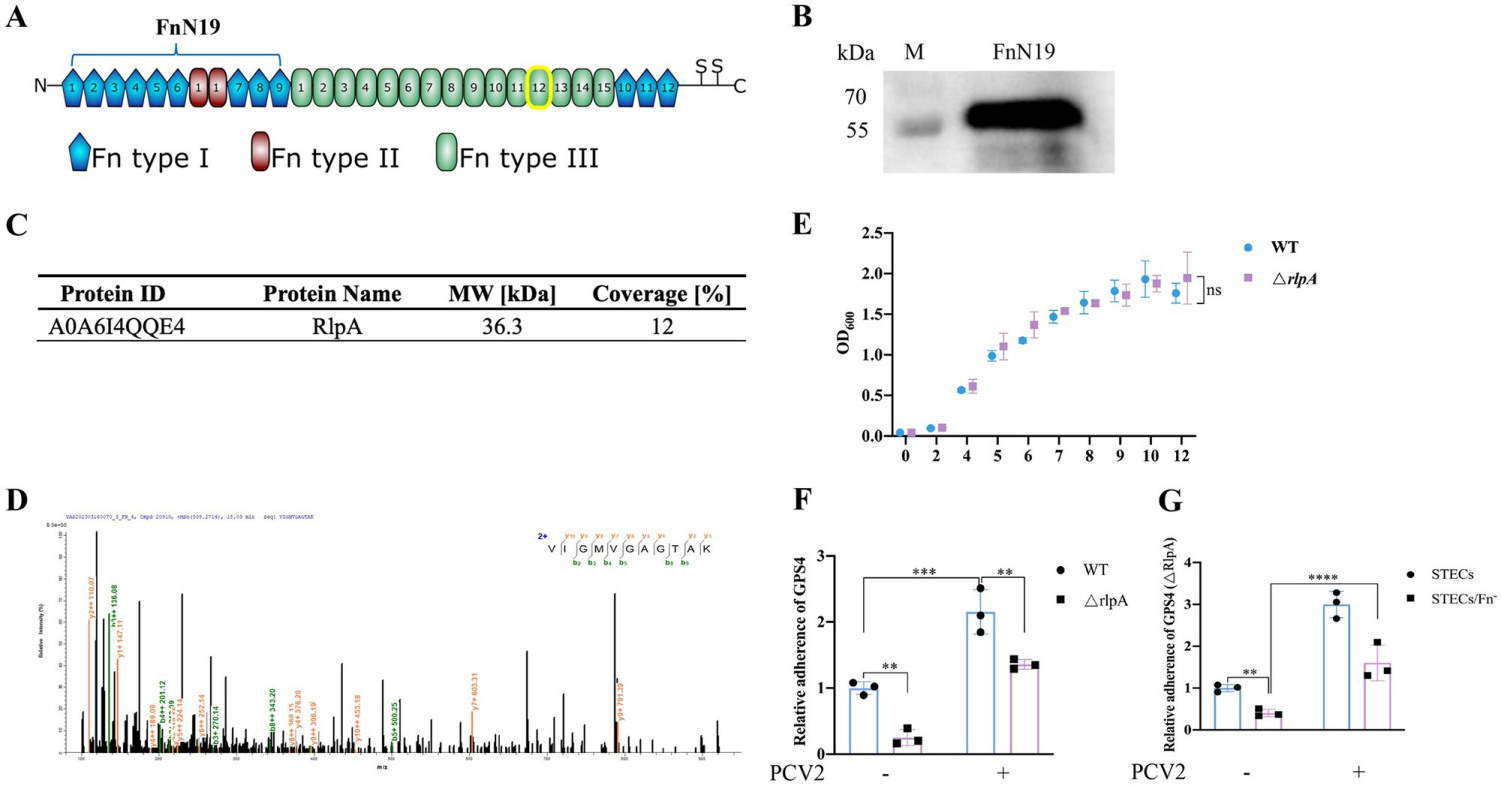
GPS4 rare lipoprotein A (rlpA) is a determinant of bacterial adherence via the Fn receptor. (A) Schematic representation of the cellular Fibronectin monomer [51]. (B) Western blot identification results of eukaryotic expression of N-terminal Fn type I and type II domains (FnN19). (C) LC-MS/MS analysis results for RlpA. (D) LC-MS/MS spectrum for the RlpA peptide. (E) Growth curve determination of wild-type strain and Δ*rlpA* deletion strain. (F) Effects of deletion of *rlpA* on GPS4 adherence. The CFUs of GPS4 in WT-infected STEC cells are considered to be 1. (G) Effect of Fn knockout on RlpA deletion strain adherence. The CFUs of △*rlpA*-GPS4 in STEC cells are considered to be 1. Data are shown as mean ± SDs of three experiments (E, F and G). Statistical analysis was performed using two-way ANOVA (E, F and G). **, *P* < 0.01; ****, *P* < 0.0001; ns, not significant.

ECM proteins and integrins are receptors that bind to microbial surface components, recognizing adhesive matrix molecules (MSCRAMM) for bacterial adherence and invasion [8,9]. The glycoprotein Fn is a multidomain protein found in various body fluids and tissues. It is mainly composed of three distinct domains, referred to as type I, type II, and type III domains [29]. In the early stage of infection, bacterial pathogens secrete a variety of virulence factors that interact with host receptors for the establishment of colonization. To identify bacterial factors responsible for Fn-mediated adherence to PCV2-infected epithelial cells, GPS4 whole bacteria proteins were reacted with recombinant His-tagged Fn N-terminal type I and type II domains (FnN19) (Fig 4A and 4B), and then Fn-binding proteins were recovered by Ni-NTA Agarose Resin 6FF for MS analysis. Four cell outer membrane proteins were identified in GPS4 lysates: Peptidoglycan-associated lipoprotein, Endolytic peptidoglycan transglycosylase RlpA, VtaA11, Virulence-associated trimeric autotransporter (S1 Table). We further investigated the RlpA, since it has previously not been reported as a bacterial adhesin. The results of RlpA liquid chromatogra-phy-tandem mass spectrometry (LC-MS/MS) analysis are shown in Fig 4C and 4D. RlpA is a lytic transglycosylase (LT) [20], predicted to be anchored to the inner leaflet of the outer membrane [21–23]. LTs are bacterial enzymes that cleave the β-1,4-glycosidic bond between sequential NAM and NAG, which are involved in cell-wall biosynthesis and recycling, cell division, insertion of cell-wall structures, and cell-wall antibiotic detection [30]. To examine whether the RlpA protein functions as an adhesin for bacterial adherence to PCV2-infected epithelial cells, we constructed *rlpA* deletion strains Δ*rlpA* (S2 Fig). Its growth rate is similar to that of wild-type strains (Fig 4E). As shown in Fig 4F, the association of wild-type (WT) strain to trachea epithelial cells was significantly higher than the adhesion activity of the *rlpA* deletion strains (Δ*rlpA*). These results suggest that GPS4 utilizes RlpA protein as an adhesin to interact with Fn on PCV2-infected cells, resulting in the establishment of a secondary GPS4 infection. In addition, our study demonstrated that there is residual Fn-mediated adhesion in Δ*rlpA* (Fig 4G).

### RlpA interacts with host Fn

**Fig 5:**
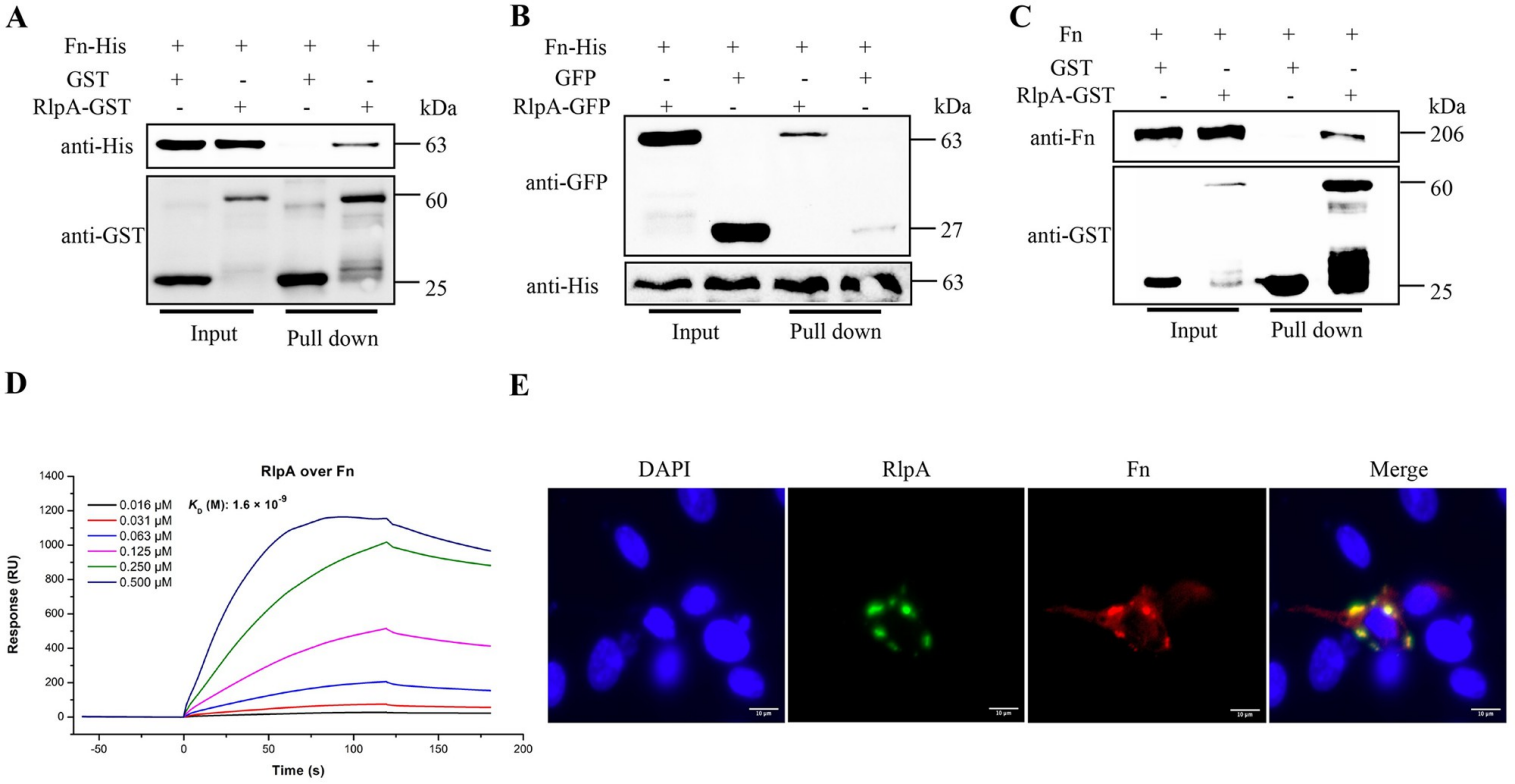
RlpA directly interacts with host Fibronectin. (A) Affinity purification of rRlpA-GST pulls down HEK293T cell lysate transfected with 7×His-FnN19-pcDNA3.1^+^, and empty GST-tag as control, GST agarose beads capture protein complexes, and the samples were detected with anti-GST and anti-His antibodies, respectively. (B) Pull-down assay confirmed the direct interaction between RlpA and Fn. RlpA-pEGFP-C3 and 7×His-FnN19-pcDNA3.1^+^ were cotransfected into HEK293T cells. The cell lysate was captured by Ni-NTA Agarose Resin 6FF beads, washed, and eluted in the sample buffer. Fractions were probed with anti-GFP and anti-His antibodies. (C) GST pull-down assay determined the interaction between RlpA and Fn in vitro. Affinity purification of rRlpA-GST pulls down rFn protein, and empty GST-tag as control, GST agarose beads capture protein complexes, and the samples were detected with anti-GST and anti-Fn antibodies, respectively. (D) Surface plasmon resonance experiment showing the dose-dependent binding profile of rRlpA (0.016–0.500 μM) over immobilized Fn. RU, response units. (E) Colocalization of the RlpA and Fn proteins was analyzed using immunofluorescence microscopy. STEC cells were cotransfected with RlpA-pEGFP-C3 and 7×His-FnN19-pcDNA3.1^+^ and labeled with Mouse anti-His-tag antibodies, followed by goat anti-mouse IgG conjugated with Alexa Fluor 647 secondary antibodies. The cells were then observed using immunofluorescence microscopy. Green represents RlpA, red represents Fn, and blue represents the nuclear stain 4′,6-diamidino-2-phenylindole (DAPI). Scale bar, 10 μm; colocalization assay suggests that RlpA is almost completely coincident with Fn. The data shown are presented as the mean ± SD of the values obtained in three independent experiments.

To confirm the interaction of Fn with the candidate bacterial RlpA protein, GST-tagged RlpA recombinant protein was reacted with lysates of HEK293T cells transfected with His-tagged FnN19, then GST pull-down assay was performed. As shown in Fig 5A, His-tagged FnN19 was pulled down by GST-tagged RlpA. To further confirm the direct interaction between RlpA and Fn, the lysates of HEK293T cells cotransfected with GFP-tagged RlpA and His-tagged FnN19 were pulled down with Ni-NTA Agarose Resin 6FF. As shown in Fig 5B, GFP-tagged RlpA was pulled down by His-tagged FnN19. As shown in Fig 5C, rFn was pulled down by rRlpA-GST. Furthermore, a surface plasmon resonance analysis confirmed the RlpA-Fn interaction, showing a dissociation constant (*K*_D_) value of 1.6×10^−9^ M (Fig 5D). Immunofluorescence showed that overexpressed GFP-tagged RlpA colocalized with Fn in STECs (Fig 5E). Additionally, our results indicate that RlpA is localized on the surface of GPS4 (S3 Fig). Taken together, these observations suggest that GPS4 adhesin RlpA binds directly to Fn on the surface of STECs.

### PCV2 infection activates the TGF-β signaling pathway and upregulates the expression of Fn to promote GPS4 adhesion in STEC

**Fig 6:**
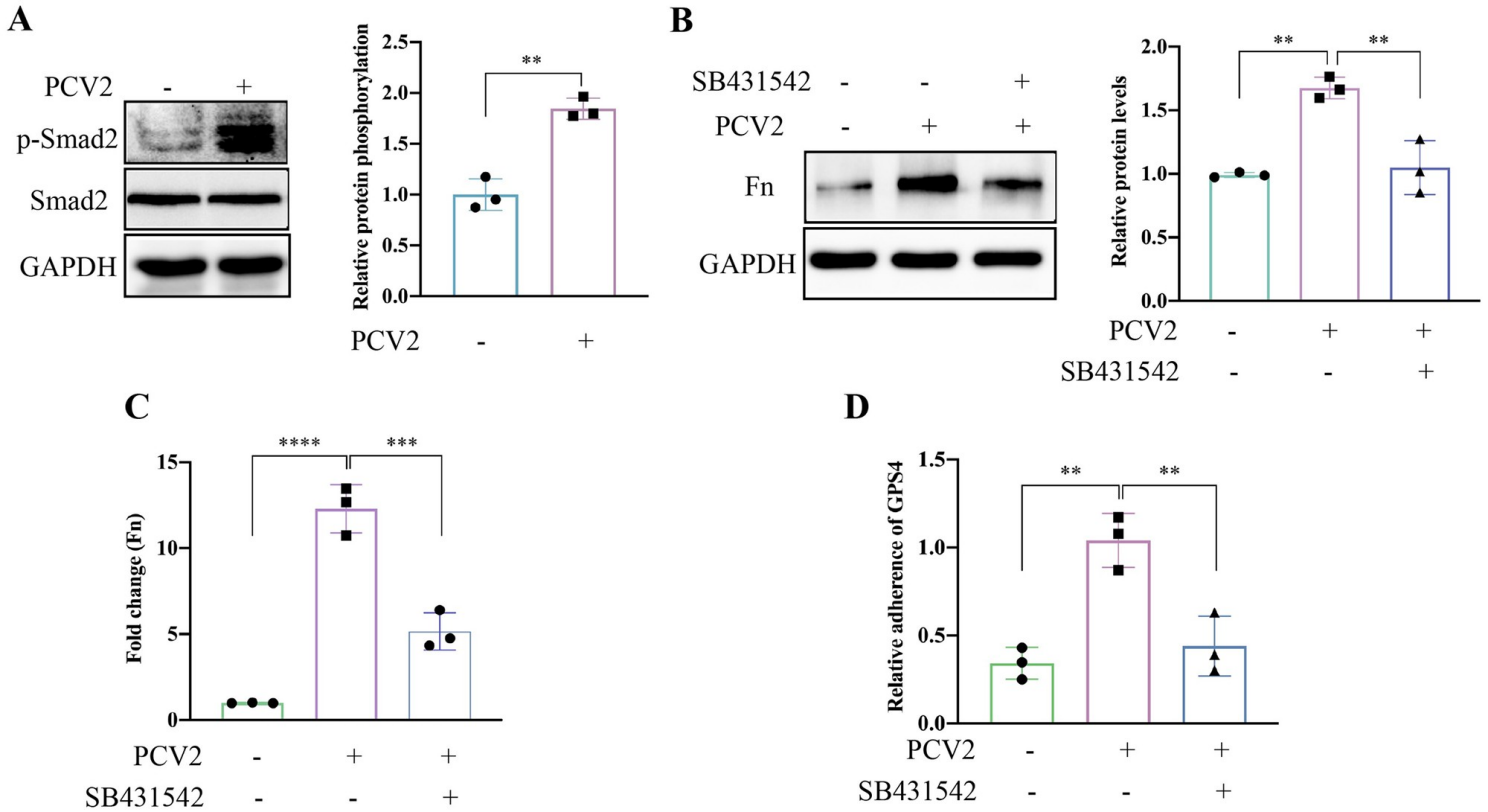
PCV2 infection-induced Fn promotes GPS4 adhesion via the TGF-β signaling pathway. STEC cells were infected with 1 MOI PCV2 virus for 36 h. (A) Smad2 phosphorylation was analyzed by Western blot. (B) The cell lysates were analyzed by Western blot for the expression of Fn using the specific antibodies in the absence or presence of 25 μM SB431542 (a TGF-β receptor kinase inhibitor). Image J software was used to analyze the intensity of protein bands, and GAPDH was used as an internal reference (A and B). (C) mRNA levels of Fn in STEC cells infected with PCV2 for 36 h in the absence or presence of 25 μM SB431542 were analyzed by RT-qPCR. (D) Colonization of GPS4 in STEC cells pretreated with 25 μM SB431542 or dimethyl sulfoxide (DMSO) for 2 h before inoculation with PCV2 (MOI = 1) or DMEM for 36 h, followed by GPS4 (MOI = 100) infection for 2 h. The CFUs of GPS4 in DMSO-treated and PCV2-infected STEC cells are considered to be 1. The data shown are presented as the mean ± SD of the values obtained in three independent experiments. Statistical analysis was performed using *t*-test (A) and one-way ANOVA (B, C, and D). **, *P* < 0.01; ***, *P* <0.001; ****, *P* < 0.0001.

Activated TGF-β can up-regulate host adhesion molecules such as fibronectin and integrins for bacterial binding [10]. We hypothesized that the activated TGF-β signaling pathway during PCV2 infection contributes to secondary GPS4 infection by up-regulating these host adhesion molecules. As shown in Fig 6A, Smad2 phosphorylation was increased in PCV2-infected STEC cells. SB431542, a TGF-β receptor kinase inhibitor, could largely counteract the expression of Fn induced by PCV2 infection (Fig 6B and 6C). Importantly, SB431542 treatment counteracted the increased GPS4 adhesion induced by PCV2 infection (Fig 6D). These results suggest that PCV2 infection-induced Fn promotes GPS4 adhesion via TGF-β signaling pathway in STECs.

## Discussion

Porcine circovirus type 2 (PCV2) can cause porcine circovirus disease and porcine circovirus-associated disease (PCVD/PCVAD), which are widespread in swine-producing countries [31,32]. Post-weaning multisystemic wasting syndrome (PMWS) of piglets caused by PCV2 is one of the major threats to the pig industry. PCV2 preferentially targets the lymphoid tissues, resulting in lymphoid depletion and immunosuppression in pigs, which often causes disease through coinfection with secondary or opportunistic pathogens, such as *Streptococcus suis* serotype 2 (SS2) [33], *Mycoplasma hyopneumoniae* and *Glaesserella parasuis* [34]. Despite antibiotic use may limit bacterial coinfections to decrease PCV2-related diseases. However, with the increase in bacterial antibiotic resistance, bacterial coinfection caused by PCV2 will inevitably become one of the most important causes of PCVAD. Therefore, understanding the underlying mechanisms of PCV2-bacterial coinfection is important for new drug development against PCV2-bacterial coinfection.

Bacterial colonization in the upper respiratory tract is considered to be a prerequisite for invasive infection, which results in bacterial invasion into other tissues or dissemination to the lower respiratory tract [12]. Interactions with host ECM components are a crucial step in the colonization and infection establishment of many bacteria, and well over 100 bacterial cell surface proteins with Fn-binding activity have been identified so far [35]. Fibronectin is a 250-kDa multidomain glycoprotein found in various body fluids and tissues. It is mainly composed of three distinct domains, referred to as type I, type II, and type III domains. The type I domain consists of 12 repeats of about 40 aa. Repeats F16 and F17 are intersected by two type II repeats (F21 and F22, each consisting of 60 aa), forming the type II domain. In total, at least 15 Fn type III repeats (each consisting of 90 aa) form the type III domain. The N-terminal type I domain and type II domain are the interaction sites with bacterial adhesins [27,36]. Among those, a predominant interaction mechanism is binding to the N-terminal Fn type I domain (F1). For example, two well-studied proteins, *S*. *aureus* FnBPA and *S*. *dysgalactiae* FnBB, bind to ^2-5^F1 and ^1-2^F1 repeats, respectively, and both proteins employ a similar tandem β-zipper mechanism [26,37]. Fibronectin, which is recognized by many bacterial pathogens, binds to membrane-spanning receptor α5β1-integrins, which mediate bacterial adhesion and invasion [38]. Previous research has shown that *S*. *pyogenes* possesses various Fn-binding molecules and utilizes Fn integrin interactions to adhere to and invade IAV-infected cells [39]. In this study, PCV2 infection upregulated the expression of ECM protein Fn and promoted the adhesion of GPS4 in tracheal epithelial cells (Figs 1 and 2). Fn knockout, Fn, and Integrin β1 antibody blocking counteract PCV2 infection-induced GPS4 adhesion (Fig 3). The above results show that PCV2 infection-induced Fn promoted the colonization of GPS4 in STEC cells.

Many bacteria express Fn-binding proteins or microbial surface components recognizing adhesive matrix molecules (MSCRAMM) [8,9]. The expression of MSCRAMM likely confers an advantage to the pre-existent bacteria in using virus-induced cellular changes for efficient colonization. Fibronectin-binding proteins (FnBPs), such as M1 and PrtF1, are crucial for GAS adherence to and subsequent entry into host cells. The capacity to bind Fn renders these bacteria capable of employing integrins as receptors for efficient internalization [19]. Nontypeable *Haemophilus influenzae* (NTHI) Haps interacts with the 45-kDa gelatin-binding domain of extracellular matrix protein fibronectin, which plays an important role in NTHI colonization of the respiratory tract [18]. *G*. *parasuis* possesses a variety of adhesins that augment bacterial adherence, such as HtrA [40], OmpP2 [41], and lipooligosaccharide (LOS) [42], but host cell receptors that interact with them have yet to be identified. Here, we identified rare lipoprotein A (RlpA), a lytic transglycosylase, as bacterial adhesin interacting with the N-terminal Fn type I and type II domains on the surface of tracheal epithelial cells following PCV2 infection (Figs 4 and 5). RlpA is a widely-conserved outer membrane protein, which is an unusual lytic transglycosylase-it preferentially digests "naked" glycan strands that lack stem peptides [20], which are involved in cell-wall biosynthesis and recycling, cell division, insertion of cell-wall structures and cell-wall antibiotic detection [23]. The rate of association of the △*rlpA* strain was decreased compared to the wild type; thus, RlpA function as an adhesin, promoting GPS4 attachment to host cells. Importantly, RlpA was first discovered as GPS4 adhesin direct interaction with Fn promoting GPS4 colonization following PCV2 infection.

FnBPs can be categorized into two groups based on their surface-anchoring mechanisms [43]. One group of FnBPs is covalently anchored to the bacterial surface. Members of this group, such as *streptococcal* fibronectin-binding protein 1 (SfbI) of *S*. *pyogenes* [44] and FnBPA of *S*. *aureus* [45], contain an N-terminal signal peptide for secretion, a C-terminal hydrophobic region, and a charged tail within which a hydrophobic domain contains the LPXTG motif for covalent anchorage to cell-wall peptidoglycan (PG). Another group of FnBPs, represented by PavA of *S*. *pneumoniae* [46] and Fbp54 of *S*. *pyogenes* [47], has been identified in many Gram-positive bacteria. Distinct from the LPXTG-mediated attachment, members of this FnBP group lack a canonical signal peptide and an LPXTG-like motif and instead use an unknown mechanism for surface localization [43]. RlpA contains an N-terminal signal peptide and a C-terminal SPOR domain, which has 50% homology with *P*. *aeruginosa* RlpA (S4 Fig). The latest research shows that *P*. *aeruginosa* SPOR-RlpA binds to PG, anchored to the inner leaflet of the outer membrane [23]. However, unlike the LPXTG-anchored FnBPs, there currently needs to be more structural and functional data regarding RlpA and how RlpA binds to Fn deserves further study.

TGF-β, a multifunctional cytokine, is secreted in an inactive or latent form and then subsequently activated through various mechanisms. During IAV infection, viral neuraminidase was shown to activate TGF-β, which promoted upregulation of host adhesion molecules, including fibronectin and integrins [19]. TGF-β is also a positive regulator of the integrin signaling pathway that promotes cytoskeletal rearrangement and bacterial internalization [10]. The inhibition of TGF-β signaling prevented adherence of GAS and other coinfecting bacteria to PR8-infected cells. Similarly, we observed that the inhibition of the TGF-β signaling pathway with SB431542 prevented the PCV2-mediated up-regulation of Fn and excess GPS4 adherence. The study demonstrated that PCV2 infection activates the TGF-β signaling pathway, which promotes the expression of Fn, leading to increased GPS4 colonization (Fig 6).

**Fig 7:**
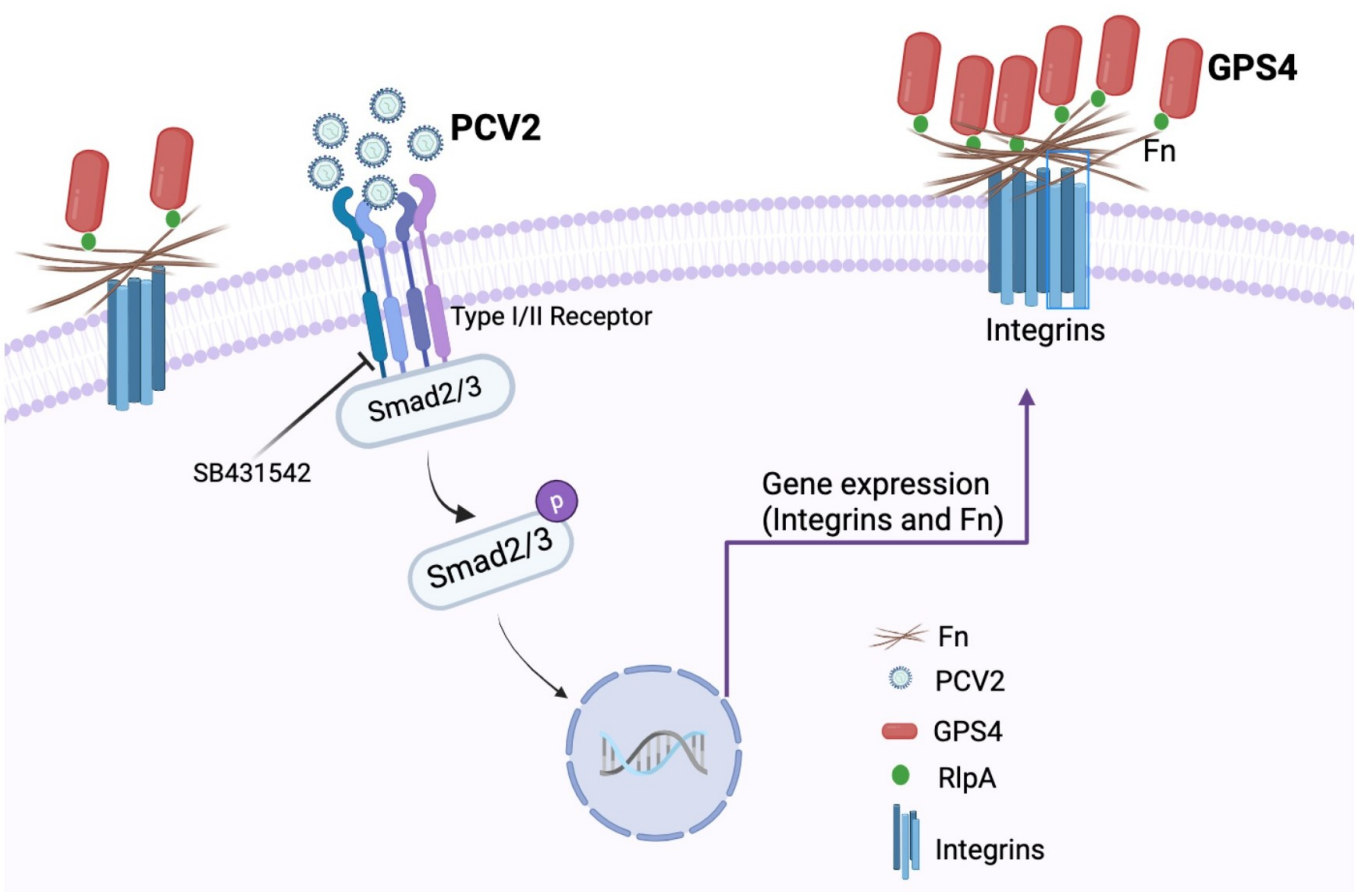
Schematic summary of PCV2 infection promotes GPS4 tracheal colonization. PCV2 infection promoted GPS4 colonization by enhancing Fn expression via the activated TGF-β signaling pathway. Importantly, GPS4 outer membrane protein RlpA directly interacts with PCV2-induced Fn to promote GPS4 tracheal colonization. SB431542 inhibitor treatment significantly inhibited GPS4 colonization in STECs after PCV2 infection.

In conclusion, this study reveals a previously unrecognized way for PCV2 to enhance bacterial coinfection. PCV2 infection promoted GPS4 colonization by enhancing Fn expression via the activated TGF-β signaling pathway. In addition, Fn deficiency, Fn and Integrin β1 antibody blocking, and SB431542 inhibitor treatment significantly inhibited GPS4 colonization in STECs after PCV2 infection. Importantly, RlpA directly interacts with PCV2-induced Fn to promote GPS4 colonization (Fig 7). These data expand the knowledge of the biological functions of RlpA in the field of bacterial infection or virus-bacterial coinfection and provide a new idea for the development of *G*. *parasuis* vaccines.

## Materials and methods

### Ethical statements

All animal experiments in this study were approved by the Animal Experimentation Ethics Committee of Nanjing Agricultural University (No2024051501) and complied with the guidelines of the Chinese Animal Welfare Committee.

### Bacterial and viral strains and culture conditions

The GPS4 strain HPS4-YC (isolated from a diseased pig in Jiangsu, China, 2016), SW124 (Standard serotype 4 strain with natural transformation ability was donated by Huazhong Agricultural University) and mutant strain were cultured in trypticase soy broth (TSB, BD, USA) or on trypticase soy agar (TSA) supplemented with 5% (v/v) FBS and 50 μg/ml nicotinamide adenine dinucleotide (NAD, Biosharp, China). All GPS4 strains were grown to the mid-log phase (an optical density at 600 nm (OD_600_) of 0.4–0.8) at 37°C, washed three times, and resuspended in DMEM unless otherwise indicated. For selection and maintenance of mutant strains, kanamycin at 50 μg/ml was added to the medium. *Escherichia coli* was grown in Luria broth (LB, Oxoid Ltd.) or LB supplemented with 1.5% agar. *E*. *coli* DH5α strain was used for cloning. *E*. *coli* BL21 (DE3) was used for protein overexpression. When necessary, kanamycin (Kan, 50 μg/ml) and ampicillin (Amp, 100 μg/ml) were added to the culture medium.

The PCV2 strain used in this study was isolated from Anhui, China, provided by the Jiangsu Academy of Agricultural Sciences (JAAS, Jiangsu, China). The virus was amplified in PK-15 cells without PCV2 infection. PK-15 cells were cultured in Dulbecco’s modified Eagle’s medium (DMEM, Gibco, Thermo Fisher Scientific) containing 5% fetal bovine serum (FBS, Invitrogen) at 37°C, 5% CO_2_.

### Cell cultures and construction of derivative STECs with lentiviruses

Swine tracheal epithelial cells (STEC) and HEK293T cells (ATCC source) were cultured in Dulbecco’s modified DMEM (Gibco, USA) supplemented with 10% (v/v) fetal bovine serum (FBS, Gibco, USA) and incubated at 37°C in an atmosphere of 5% CO_2_.

Three pairs of sgRNAs targeting the porcine Fn genome sequence were cloned into the lentiCRISPR v2 vector (Addgene, USA), digested with BsmBI (NEB, R0739) and cotransfected with pCMV-VSV-G (Addgene) and psPAX2 (Addgene) into HEK293T cells for lentivirus packaging. STECs were infected with lentiviruses at an MOI of 1:1 for 36 h. The on-target efficiency was analyzed by Western blot. The on-target was screened in the presence of puromycin (3 μg/ml, Beyotime) for 7 d. Western blot was used to determine the expression of Fn.

### Adherence assay

The STEC cells were seeded in 24-well plates and incubated at 37°C, 5% CO_2_ incubator until the cells became confluent. They were divided into the PCV2-infected group and the noninfected group. After 24h, 36 h and 48h of PCV2 (1 MOI) infection, both groups were infected with GPS4 at MOI 100. The cell plate was centrifuged at 800 × *g* for 10 min, incubated at 37°C 5% CO_2_ incubator for 2 h, and washed three times with phosphate buffer saline (PBS) to remove non-adherent bacteria. For adhesion experiments, cells were lysed with sterile double distilled water for 10 min and diluted with PBS. The number of adherent bacteria was determined by spreading on supplemented TSA plates.

In some experiments, antibodies targeting Fn (rat polyclonal antibody; BOSTER), anti-Integrin β1 antibody (rat MAb; ab179471), and rat IgG isotype control (rat MAb; R&D) were added to PCV2-infected 36 h STEC cells for 3 h before infection with GPS4. Assays were performed in triplicate and repeated three times.

### Plasmids construction and preparation of recombinant proteins

For the expression of GST-tagged protein, r*lpA* devoid of the signal peptide-encoding sequence was cloned into the BamH I and EcoRI sites of pGEX-4T-1 and then transformed into *E*. *coli* BL21(DE3). For the expression of proteins in eukaryotic cells, *rlpA* devoid of the signal peptide-encoding sequence was cloned into the Xho I and BamH I sites of pEGFP-C3; the N-terminal Fn type I and type II domain (FnN19) was amplification from Sus scrofa fibronectin 1 (XM_003133642.5) and cloned into the BamH I and EcoRI sites of 7×His-pcDNA3.1^+^. All plasmid constructs were propagated in *E*. *coli* strain DH5α. The primers used in this study were listed in S2 Table, and the strains and plasmids used in this study were listed in S3 Table.

### Isolation and identification of cell surface proteins

Cell surface proteins were biotinylated and labeled with Thermo Scientific Z-Link Sulfo-NHS-SS-Biotin, a thiol cleavable amine-reactive biotinylation reagent according to the manufacturer’s protocol (A44390). Briefly, noninfected and PCV2-infected STEC cells were washed with PBS and then incubated with sulfo-NHS-biotin for 10 min at room temperature. Cells are subsequently lysed with detergent for 30 min on ice and the labeled proteins are then isolated with Thermo Scientific NeutrAvidin Agarose for 30 min at room temperature. The bound, labeled proteins are released by reduction of the disulfide bond with 10 mM DTT for 30 min at room temperature. The prepared protein samples were subjected to mass spectrometry (MS) analysis (Shanghai Applied Protein Technology Co., Ltd., China).

### Pull-down assay

For identification of bacterial factors associated with Fn, constructed 7×His-FnN19 pcDNA3.1^+^ was transfected into HEK293T cells using Lipofectamine 2000 (Invitrogen) for large-scale expression. GPS4 whole bacterial proteins were incubated with recombinant FnN19 in buffer A (20 mM Tris, pH 8.0, 150 mM NaCl, 0.1% Triton X-100) for 6 h at 4°C. Proteins bound to FnN19 were pulled down with Ni-NTA Agarose Resin 6FF (solarbio). Ni-NTA Agarose Resin 6FF was used as a control. After incubation, the beads were collected by centrifugation at 500 × *g*, 5 min, and 4°C and washed five times with buffer A. Then, the beads were resuspended in 20 μl SDS sample buffer and boiled for 10 min. Retained proteins were separated using SDS-PAGE after 10% SDS gel and then examined using mass spectrometry analysis.

To test the interaction between RlpA and FnN19, rRlpA-GST (r means recombinant protein) was incubated with BeyoGold GST-tag Purification Resin for 1 h at 4°C and incubated with HEK293T cell lysate overexpressing FnN19 and purified 206 kDa rFn (YEASEN, 40113ES03) for another 6 h at 4°C. As previously described [48], an empty GST tag was used as a control. After incubation, the beads were collected by centrifugation at 500 × *g*, 5 min, and 4°C and washed five times with buffer A. Then, the beads were resuspended in 20 μl SDS sample buffer and boiled for 10 min. Retained proteins were detected by Western blotting after 10% SDS gel.

RlpA-pEGFP-C3 and 7×His-FnN19-pcDNA3.1^+^ were cotransfected into HEK293T cells. At 48 h after transfection, the cells were lysed with IP buffer containing protease inhibitors and phosphatase inhibitors (Beyotime) and centrifuged at 13,000 × *g* at 4°C for 10 min, pEGFP-C3 and 7×His-FnN19-pcDNA3.1^+^ cotransfected HEK293T cells as control. The resulting supernatants were pulled down with Ni-NTA Agarose Resin 6FF at 4°C for 2 h. After incubation, the beads were collected as described above for Western blot analysis.

### Surface plasmon resonance analysis

Surface plasmon resonance experiments were performed at 25°C using a Biacore T200 system (GE Life Sciences, USA). rFn was immobilized on the CM5 sensory chip at 8000 response units (RUs). An uncoated "blank" channel was used as a negative control. All materials were exchanged to or dissolved in Hepes buffer [10 mM Hepes (pH 7.4), 150 mM NaCl, 3 mM EDTA, and 0.05% P20]. rRlpA was diluted in running buffer and injected at different concentrations at a flow rate of 30 μl/min at 25°C. The kinetic parameter analyses were performed using Biacore T200 Evaluation Software with a 1:1 Langmuir binding model.

### Western blots

Electrophoretically separated proteins were transferred to polyvinylidene fluoride (PVDF) membranes (Millipore, USA), which were then blocked with 3% bovine serum albumin (BSA) for 2 h. The membranes were then incubated with primary antibodies at 4°C overnight. The primary antibodies used were as follows: anti-Fn rabbit polyclonal antibody (1:1000, BA1772) was purchased from BOSTER (Wuhan, China); anti-GFP Mouse mAb (1:5000, M20004) was purchased from Abmart (Shanghai, China); Mouse anti-His-tag antibodies (1:1000, AT0025), Mouse anti-GST-tag antibodies (1:1000, AT0098) and anti-GAPDH mouse mAb (1:5,000; AT0002) were purchased from Engibody Biotechnology (Milwaukee, WI, USA); Smad2 rabbit monoclonal antibody (1:1000, 5339) and phospho-Smad2 rabbit monoclonal antibody (1:1000, 18338) were purchased from Cell Signaling Technology (Beverly, MA, USA).

The membranes were washed with TBST and then incubated with HRP-conjugated goat anti-rabbit (1:5000; Cat. No. AT0097, Engibody Biotechnology) or goat anti-mouse IgG antibody (1:5000; Cat. No. AT0098, Engibody Biotechnology) at room temperature for 45 min. The membranes were incubated with ECL Femto-Detect Western Blotting Substrate (Engibody Biotechnology) and exposed using a ChemiDoc Touch Imaging System (Bio-Rad), and the resulting images were analyzed using Image Lab software. The images shown are representative of three independent experiments.

### Quantitative RT-PCR

STECs were lysed with TRIzol (Vazyme Biotech Co., China), and total RNA was isolated according to the protocol provided by the manufacturer. cDNA was synthesized using HiScript Q RT SuperMix for qPCR (+gDNA wiper) (Vazyme Biotech Co.). qRT-PCR was performed on StepOne real-time fluorescent quantitative systems (Applied Biosystems, ABI StepOne) with ChamQ Universal SYBR qPCR Master Mix (Vazyme Biotech Co.). The primer sequences are shown in S2 Table. The *GAPDH* gene was used as an internal control, and relative quantification compared to the uninfected cells was calculated using the 2^-ΔΔCt^ method [49]. The tests were performed in triplicate and each set of qPCR assays was repeated three times.

### siRNA transfection

si-FN1-Sus-7815 (sense: 5’-GCCCAAUUGAGUGCUUCAUTT-3’; antisense: 5’-AUGAAGCACUCAAUUGGGCTT-3’) was synthesized by Shanghai GenePharma and used to knock down the expression of Fn in STEC cells. When the STEC cell density reached 30%-50%, the diluted si-FN1 and Lipofectamine 2000 (Invitrogen, 11668019) were incubated in serum-free DMEM for 15 min at room temperature, and the working concentration of si-FN1 was 50 nM for transfection for 12 h. PCV2 and GPS4 infection was then performed.

### Construction of the △*rlpA* mutant

The oligonucleotide primers used for PCR are listed in S2 Table. The 528 bp upstream and 509 bp downstream fragments of the *rlpA* gene were amplified from the HPS4-YC genome using primer pairs *rlpA*-up-F/R and *rlpA*-down F/R, respectively. These two sets of primers contained a 9-bp core DNA uptake signal sequence (USS) (ACCGCTTGT) required for the natural transformation of *G*. *parasuis*. A 933 bp kanamycin resistance cassette was amplified from pK18mobsacB with primers kan-F/R. These three fragments were linked with overlap extension PCR using the primers *rlpA*-up-F and *rlpA*-down-R. The PCR product (EcoR I and BamH I) and pK18mobsacB digested by EcoR I and BamH I and then ligated together to generate the recombinant plasmid pK18-Δ*rlpA*::kan. Subsequently, the plasmid was introduced into *G*. *parasuis* standard serotype 4 strain SW124 by natural transformation, as described previously [40]. Briefly, 20 μl of cAMP (8 mM) was added to 20 μl of recipient bacterial suspension in the logarithmic phase (optical density at 600 nm (OD_600_) of approximately 0.9). Next, 2 μg of donor DNA plasmid was added to the bacterial mixture, which was then spotted onto a TSA plate, spread in a small area, and incubated at 37°C for 5 h. Ultimately, bacterial cells were collected, plated on a kanamycin-selective medium, and incubated at 37°C for 48 h.

Growth curve measurement: cultured indicated bacteria were diluted OD_600_ to 0.01–0.02, and the OD_600_ was measured manually every 2 h for 12 h.

### Immunofluorescence microscopy

To further demonstrate the colocalization of RlpA and Fn, RlpA-pEGFP-C3 and 7×His-FnN19-pcDNA3.1^+^ were cotransfected into STEC cells. Indirect immunofluorescence staining of transfected cells was performed at 48 h after transfection as described previously [50]. Briefly, transfected STEC cells were fixed with ice-cold methanol for 20 min, permeabilized with PBS containing 0.1% Triton X-100 for 5 min, and then blocked with 3% BSA at 37°C for 1 h. The samples were incubated overnight at 4°C with the Mouse anti-His-tag antibodies (1:1000, AT0025). Then the samples were incubated with goat anti-mouse IgG conjugated with Alexa Fluor 647 (1:200, Abcam, ab150115) for 1 h in a 37°C incubator. Cell nuclei were stained with DAPI.

To confirm RlpA surface localization on GPS4, GPS4 and *△rlpA* mutant were grown exponentially in TSB broth, and 1 ml bacterial cells were collected by centrifugation at 2000 × *g*, at room temperature for 10 min. Then, washed two times with sterile PBS. Subsequently, cells pellets were resuspended in 100 μl PBS and 5 μl transferred onto slides, air dried, fixed with 4% paraformaldehyde (Sigma-Aldrich) (pH 7.5) by incubating for 15 min at room temperature, rinsed three times with PBS, and allowed to air dry again. Then, 200 μl mouse anti-RlpA serum was added at a 1:100 dilution to cover all cell areas, and the mixture was incubated at room temperature for 90 min, washed three times with PBS, and RlpA was detected by adding 200 μl goat anti-mouse IgG conjugated with Alexa Fluor 647 (Abcam, ab150115) at a 1:200 dilution and incubated at room temperature for 30 min. For DAPI, 100 μl DAPI was added and incubated for 10 min at room temperature. After a last wash with PBS, the slides air dried, and then 5μl mounting medium was added and covered by a cover slip. Finally, all samples were observed and imaged with a Zeiss laser scanning microscope (Carl Zeiss, Germany).

### Inhibition assays

To inhibit the TGF-β signaling pathway, PCV2-infected STEC cells were treated with 25 μM SB431542 (a TGF-β receptor kinase inhibitor). For the controls, equal volumes of DMSO were added to the cell culture.

### Protein sequence alignment and analysis

The analysis of RlpA signal peptide was predicted by SignalP-5.0 (https://services.healthtech.dtu.dk/services/SignalP-5.0/) online. The RlpA protein sequence was aligned against all homology sequences by the blastp (blast.ncbi.nlm.nih.gov), and the selected corresponding homology sequences from *E*. *coli*, *P*. *aeruginosa*, *H*. *influenzae*, and *Neisseria* were aligned using Clustal Omega (ebi.ac.uk/Tools/msa/clustalo) and presentation of alignment was performed using software Jalview with conserved [48].

### Statistical analysis

All experiments were independently repeated at least three times. Data are presented as mean ±SDs, and GraphPad Prism 8 was used for one-way analysis of variance (ANOVA), two-way ANOVA, and an unpaired *t*-test. *P* < 0.05 was considered statistically significant, ns: no significant.

## Supporting information

S1 Fig Establishment of the Fn KO STEC cell line. (A) Construction of LentiCRISPRv2 vector for Fn knockout. M: DL2000 DNA Marker; 1–6: Detection of sgFn-LentiCRISPRv2 plasmid. (B) Western blot detects the expression of Fn in STEC infected with lentivirus, and GAPDH was used as an internal reference.
(TIF)

S2 Fig PCR identification of Δ*rlpA*. Δ*rlpA* was identified by PCR using primers *rlpA* gene and *kana* gene. M: DL2000 DNA Marker.
(TIF)

S3 Fig Surface localization of RlpA on GPS4 using immunofluorescence microscopy. Fixed GPS4 and △*rlpA* were labeled with mouse anti-RlpA antibodies, followed by goat anti-mouse IgG conjugated with Alexa Fluor 647 secondary antibodies. Red represents RlpA, and blue represents DAPI. Scale bar, 2 μm.
(TIF)

S4 Fig RlpA signal peptide and conservation analysis. (A) RlpA signal peptide analysis. (B) RlpA conservation analysis.
(TIF)

S1 Table Mass Spectrometry results of cell outer membrane proteins pulled down with recombinant FnN19 from GPS4 lysates. (DOCX)

S2 Table Primers used in this study. (DOCX)

S3 Table Strains and plasmids in this study. (DOCX)

S1 Data Excel spreadsheet containing the numerical data and statistical analysis for Fig panels 1, 2D, 3B-C, E-G, 4E-G, 5D, 6C-D. (XLSX)
