# Supplementary figures and images for: Glaesserella parasuis serotype 4 exploits fibronectin via RlpA for tracheal colonization following porcine circovirus type 2 infection
Source: PLoS Pathog. 2024 Sep 12;20(9):e1012513. doi: 10.1371/journal.ppat.1012513 (PMC11392263; doi:10.1371/journal.ppat.1012513)

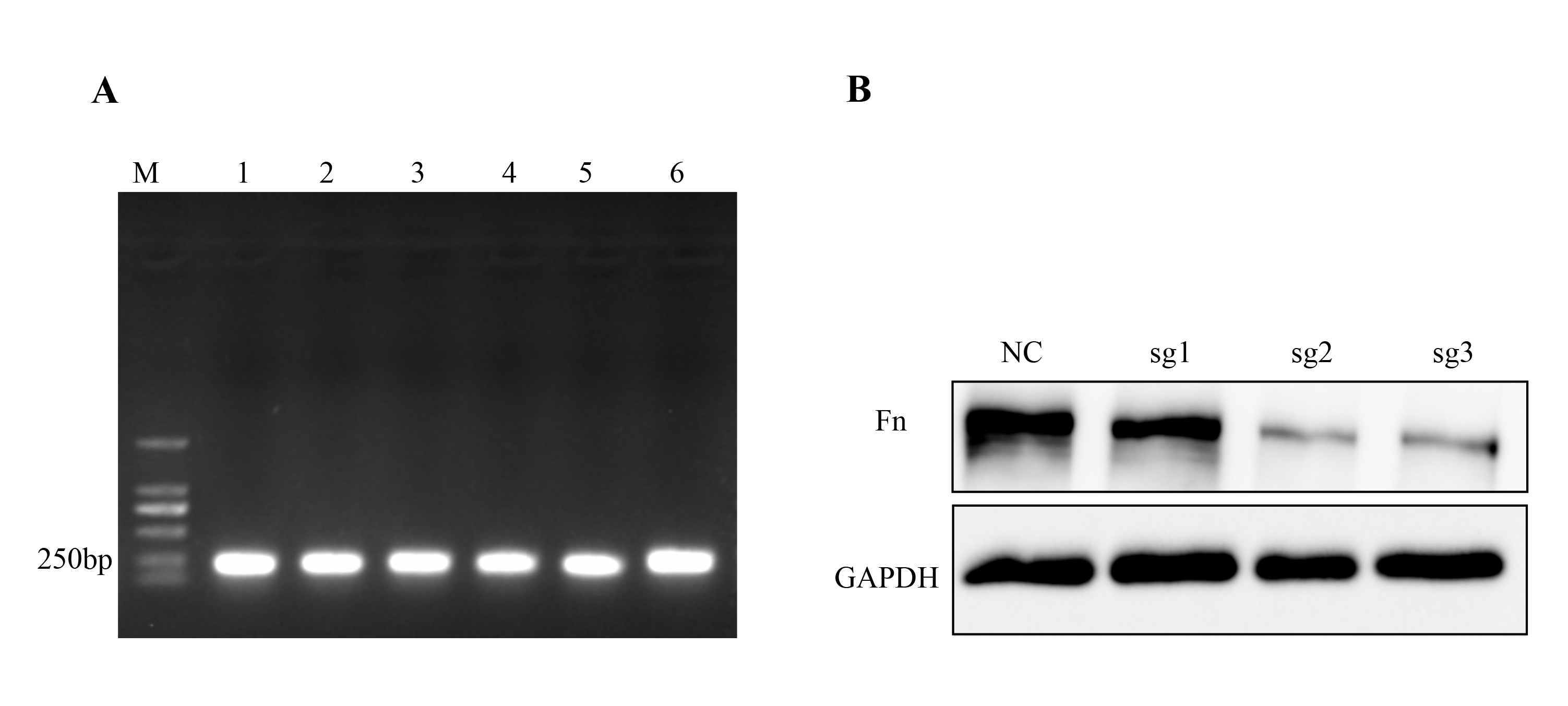

Supplement: S1 Fig — (A) Construction of LentiCRISPRv2 vector for Fn knockout. M: DL2000 DNA Marker; 1–6: Detection of sgFn-LentiCRISPRv2 plasmid. (B) Western blot detects the expression of Fn in STEC infected with lentivirus, and GAPDH was used as an internal reference. (TIF) [file ppat.1012513.s001.tif]

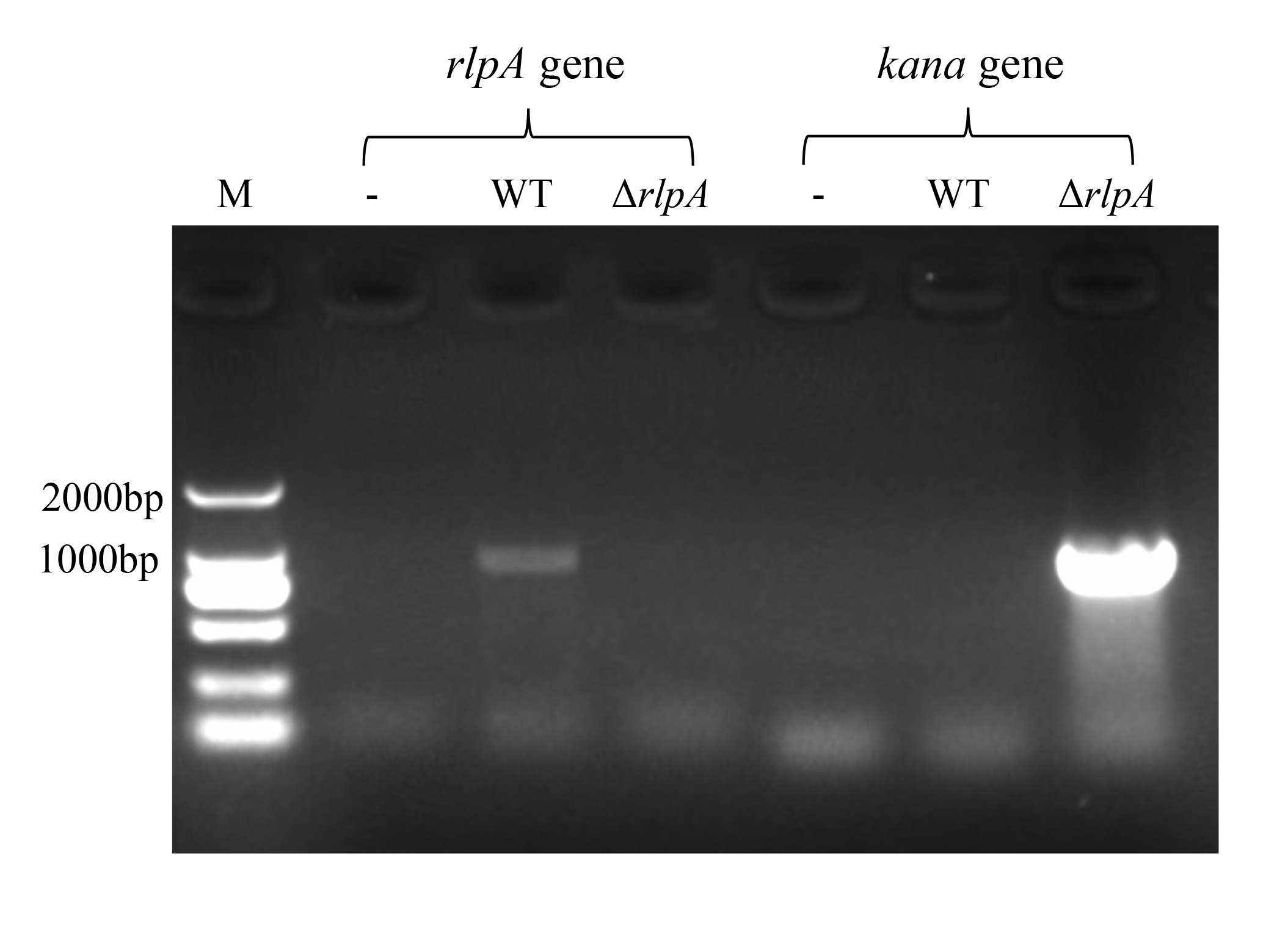

Supplement: S2 Fig — ΔrlpA was identified by PCR using primers rlpA gene and kana gene. M: DL2000 DNA Marker. (TIF) [file ppat.1012513.s002.tif]

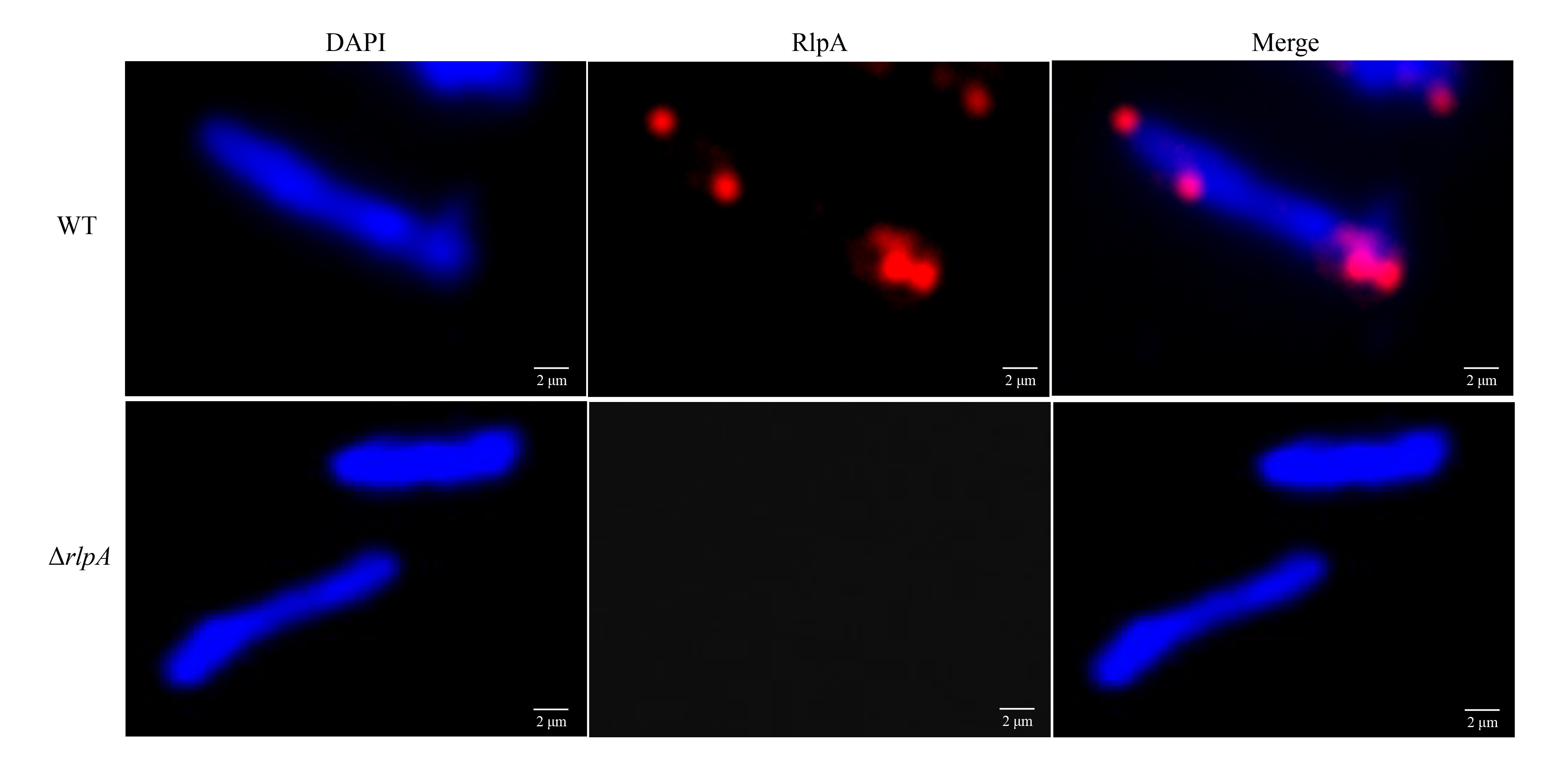

Supplement: S3 Fig — Fixed GPS4 and △rlpA were labeled with mouse anti-RlpA antibodies, followed by goat anti-mouse IgG conjugated with Alexa Fluor 647 secondary antibodies. Red represents RlpA, and blue represents DAPI. Scale bar, 2 μm. (TIF) [file ppat.1012513.s003.tif]

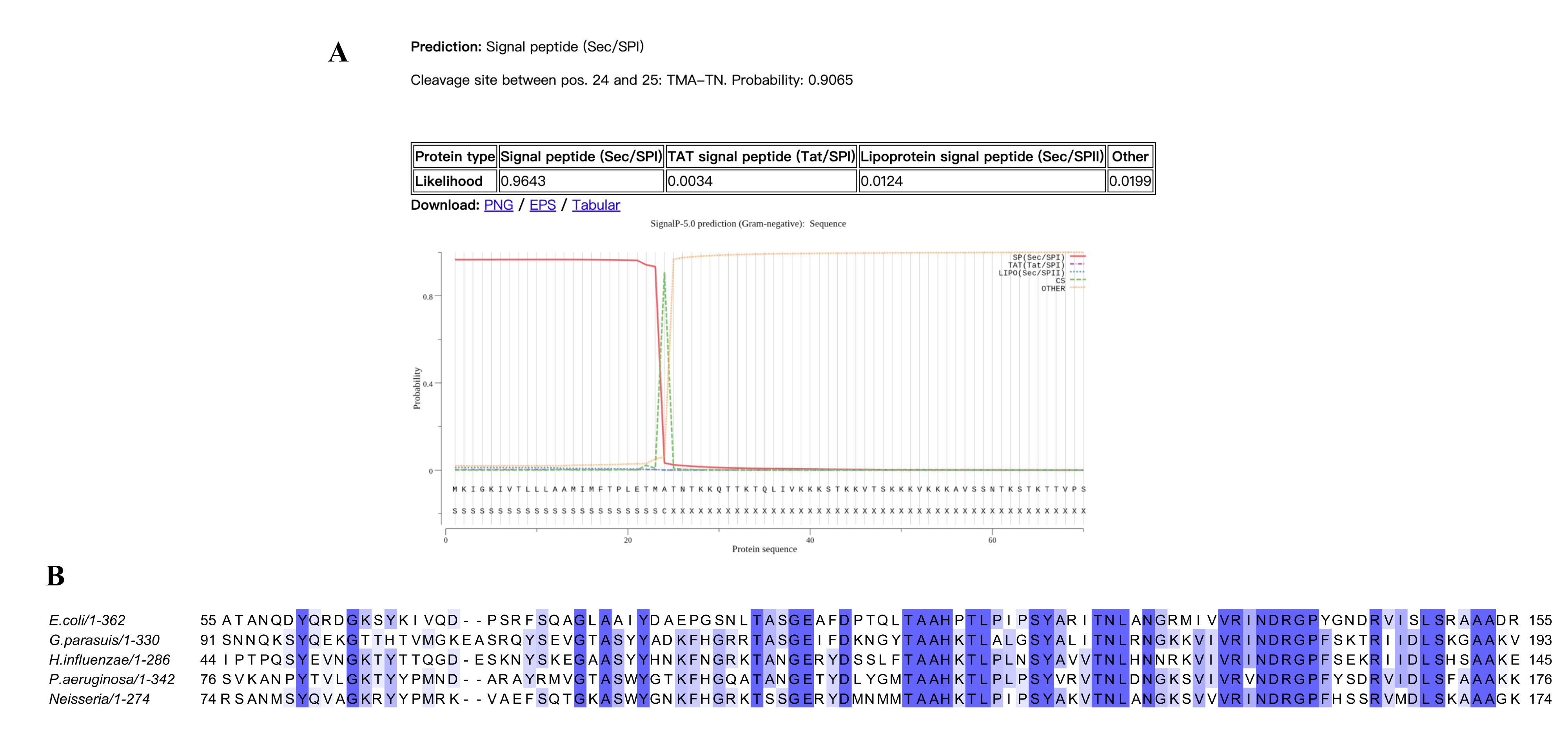

Supplement: S4 Fig — (A) RlpA signal peptide analysis. (B) RlpA conservation analysis. (TIF) [file ppat.1012513.s004.tif]
