# Supplementary material for: Glaesserella parasuis serotype 4 exploits fibronectin via RlpA for tracheal colonization following porcine circovirus type 2 infection
Source: PLoS Pathog. 2024 Sep 12;20(9):e1012513. doi: 10.1371/journal.ppat.1012513 (PMC11392263; doi:10.1371/journal.ppat.1012513)
Supplement: S1 Table — (DOCX) [file ppat.1012513.s005.docx]

**S1 Table. Mass Spectrometry results of cell outer membrane proteins pulled down with recombinant FnN19 from GPS4 lysates.**

| **Accession** | **Description** | | **Coverage [%]** | **#Peptides** | **MW [kDa]** |
| --- | --- | --- | --- | --- | --- |
| A0A8A2WD94 | Peptidoglycan-associated lipoprotein  Endolytic peptidoglycan transglycosylase RlpA  VtaA11  Virulence-associated trimeric autotransporter | | 44 | 4 | 16.3 |
| A0A6I4QQE4 |  |  | 12 | 3 | 36.3 |
| B8QR27  F2GDD9 |  |  | 6  4 | 6  4 | 150.3  132.5 |
|  |  |  |  |  |  |
