# Supplementary material for: Glaesserella parasuis serotype 4 exploits fibronectin via RlpA for tracheal colonization following porcine circovirus type 2 infection
Source: PLoS Pathog. 2024 Sep 12;20(9):e1012513. doi: 10.1371/journal.ppat.1012513 (PMC11392263; doi:10.1371/journal.ppat.1012513)
Supplement: S2 Table — (DOCX) [file ppat.1012513.s006.docx]

**S2 Table. Primers used in this study**

| **Primers** | **Sequence (5’-3’)** |
| --- | --- |
| **For strains construction** | |
| *rlpA*-up-F | ctatgacatgattacgaattc *ACCGCTTGT﻿*ATGACGTTAATTGATCCCG |
| *rlpA*-up-R | gcagggcttcccaaccttac ﻿TAAATTCCCCTTTTAATC |
| *rlpA*-down-F | ggggttcgctagaggatc ﻿ATAGGATTTTTTATATGTT |
| *rlpA*-down-R | caggtcgactctagaggatcc *ACAAGCGGT*﻿﻿ATGCGTATTTTTCAAACC |
| *kana-F* | gtaaggttgggaagccctgc |
| *kana-R* | gatcctctagcgaacccc |
| **Prokaryotic expression** | |
| pGEX4T-1-*rlpA*-F | TCCAGGGGCCCCTGGGATCC ﻿acaaatacaaagaaacaaac |
| pGEX4T-1-*rlpA*-F | TCGAGTCGACCCGGGAATTC ﻿ctatttttctgaataactg |
|  | **For Fn knockout** |
| gF1-F | CACCgagtctgccgtacagtccaa |
| gF1-R | AAACttggactgtacggcagactc |
| gF2-F | CACCGcaggccaatcggcgagcggt |
| gF2-R | AAACaccgctcgccgattggcctgC |
| gF3-F | CACCgccaatcggcgagcggtcgg |
| gF3-R | AAACccgaccgctcgccgattggc |
| CRISPRv2-R | CTAGGCACCGGATCAATTGC |
| **For RT-qPCR** | |
| Fn-F(qPCR) | GTGACAGATGCTACTGAGAC |
| Fn -R(qCR) | TCTCTGGATTGGAGTCTGGC |
| GAPDH-F(qPCR) | GATGCTGGTGCTGAGTATGT |
| GAPDH-R(qPCR) | GGCAGAGATGATGACCCTTT |
| **Eukaryotic expression** | |
| 7×His-pcDNA3.1^+^ Fn*-*F | GCTTGGTACCGAGCTCGGATCC GCCACC ATG TGTTACGACAATGGAAAACAC |
| 7×His-pcDNA3.1^+^ Fn-R | TGGATATCTGCAGAATTC TTAATGGTGATGGTGATGATGATG GGTCTGTAAGGGTTGGC |
| pEGFP-C3- *rlpA*-F | tacaagtactcagatctcgag acaaatacaaagaaacaaac |
| pEGFP-C3- *rlpA*-R | ttatctagatccggtggatcc ﻿﻿ctatttttctgaataactg |
