# Supplementary material for: Glaesserella parasuis serotype 4 exploits fibronectin via RlpA for tracheal colonization following porcine circovirus type 2 infection
Source: PLoS Pathog. 2024 Sep 12;20(9):e1012513. doi: 10.1371/journal.ppat.1012513 (PMC11392263; doi:10.1371/journal.ppat.1012513)
Supplement: S3 Table — (DOCX) [file ppat.1012513.s007.docx]

**S3 Table. Strains and plasmids used in this study.**

| **Name** | **Characteristics** | **Source** |
| --- | --- | --- |
| **Strains** | | |
| YC1601 | Isolated from a diseased pig in Jiangsu, China | Lab stocks |
| SW124 | Donated by Huazhong Agricultural University | Lab stocks |
| Δ*rlpA* | Isogenic *rlpa* mutant of SW124, Kan^R^ | This study |
| *E. coli*DH5α | Cloing host for maintaining the recombinant plasmids | Lab stocks |
| *E. coli* BL21 | The expression host of recombinant proteins | Lab stocks |
| **Plasmids** | | |
| pGEX4T-1 | Prokaryotic expression | Lab stocks |
| pcDNA3.1^+^ | Eukaryotic expression | Lab stocks |
| pEGFP-C3 | Eukaryotic expression | Lab stocks |
| pGEX4T-1-*rlpA* | Cloning expression of recombinant rlpA, Amp^R^ | This study |
| 7×His-pcDNA3.1^+^ Fn | Cloning expression of recombinant Fn, Amp^R^ | This study |
| pEGFP-C3- *rlpA* | Cloning expression of recombinant rlpA, Kan^R^ | This study |
| pK18mobsacB | Suicide and narrow-broad-host vector, Kan^R^ | Lab stocks |
| LentiCRISPRv2 | Lentiviral CRISPR/Cas9 knock-out vector system | Lab stocks |
| pCMV-VSV-G | Lentiviral packaging plasmid | Lab stocks |
| psPAX2 | Lentiviral packaging plasmid | Lab stocks |
